# Impact of metagenomic next-generation sequencing on clinical decision-making at an academic medical center, a retrospective study, Iowa, 2020–2022

**DOI:** 10.1017/ash.2024.31

**Published:** 2024-03-27

**Authors:** Michael Olthoff, Takaaki Kobayashi, Meredith G. Parsons, Bradley Ford, Kunatum Prasidthrathsint, Lemuel Non, Jorge L. Salinas, Daniel J. Diekema, Dilek Ince

**Affiliations:** 1 Department of Internal Medicine, University of Iowa Hospitals and Clinics, Iowa City, IA, USA; 2 Carver College of Medicine, University of Iowa, Iowa City, IA, USA; 3 Department of Pathology, University of Iowa Hospitals and Clinics, Iowa City, IA, USA; 4 Department of Internal Medicine, Stanford University, Stanford, CA, USA; 5 Department of Medicine, Maine Medical Center, Portland, ME, USA

## Abstract

We assessed the impact of metagenomic next-generation sequencing (mNGS) on patient care using previously established criteria. Among 37 patients receiving mNGS testing, 16% showed results that had a positive clinical impact. While mNGS results may offer valuable supplementary information, results should be interpreted within the broader clinical context and evaluation.

## Introduction

Metagenomic next-generation sequencing (mNGS) has the potential to identify a variety of pathogens from a single blood sample, to aid in the diagnosis of deep-seated and bloodstream infections. One commercially available assay, the Karius test, reportedly detects 1,250 species of pathogens.^1^ Sensitivity (51–90%) and specificity (86%) of mNGS have been reported to outperform culture in prior studies.^2,3^ However, there are known limitations to mNGS, particularly identification of genetic material from transiently translocated organisms, the current high cost of testing, and lack of antimicrobial susceptibility results. Furthermore, only a few studies have evaluated the impact of mNGS on clinical decision-making when compared to more conventional diagnostic methods.^4,5^ In this study we assess the clinical impact of the Karius test on patient care at an Iowa Academic Medical Center.

## Methods

We conducted a retrospective cohort study of patients at a single center, the University of Iowa Hospitals & Clinics, spanning from January 2020 to June 2022. All patients who underwent mNGS testing with the Karius assay were included in this review. Any provider has the option to request Karius testing, but approval from a microbiology director is necessary, and the testing is authorized only when conventional tests, like blood culture and biopsies, yield negative results or are infeasible. A team of infectious disease physician reviewers (MO, TK, DI, KP, LN) conducted a comprehensive chart review focusing on patient characteristics, clinical progression, and outcomes. The results of mNGS and conventional tests were compared for concordance. Conventional tests encompassed cultures, serology, antigen testing, and targeted polymerase chain reaction (PCR). We assessed the clinical impact of mNGS testing using criteria established in prior studies.^4^ These were classified into positive impact, negative impact, no impact, or indeterminate (Supplementary Table 1 (online)). This study was approved by the institutional review board.

## Results

A total of 37 patients had the Karius test performed during our study window. Fever was the most commonly observed symptom in 65% of patients, followed by respiratory symptoms at 57%. Among these 37 patients, 29 (78%) had an immunocompromising condition, with the most common being hematologic malignancy (21 patients, 58%, as shown in Table 1). Additionally, 34 patients (92%) had received a formal infectious disease (ID) consultation prior to the performance of the Karius test. The most common finding before testing was the presence of pulmonary infiltrates on imaging (30 patients, 81%). Thirteen patients had positive results for both mNGS and conventional testing, of which 12 had completely or partially identical organisms. The Karius test results were determined to have a positive clinical impact in six cases (16%, Table 2, Supplemental Table 1 (online)), an indeterminate impact in one case (3%), and no impact in 30 cases (81%).

**Table 1.** Patient demographics, concordance, and impact of metagenomic next-generation sequencing

Total patients	*n* = 37
Age (years), median (+/− SD)	54.3 (17.8)
Sex	
Female	14 (43%)
Male	23 (58%)
Clinical manifestation	
Fever	24 (65%)
Respiratory symptoms	21 (57%)
Gastrointestinal symptoms	7 (19%)
Mental status change	6 (16%)
Rash	4 (11%)
Musculoskeletal symptoms	4 (11%)
Immunocompromising condition	
Bone marrow transplant	11 (30%)
Solid organ transplant	4 (11%)
Hematologic malignancy	21 (58%)
Presence of pulmonary infiltrates/nodules	30 (81%)
Infectious disease consult	34 (92%)
Purpose of testing	
Diagnosis	30 (81%)
Rule out	5 (14%)
Unknown	2 (5%)
Concordance with conventional testing	
Both mNGS and conventional testing positive	13 (35%)
Completely same organism(s)	4 (31%)
Partially same organisms(s)	8 (22%)
Different organism(s)	1 (27%)
mNGS positive but conventional testing negative	13 (35%)
mNGS negative but conventional testing positive	1 (3%)
Both mNGS and conventional testing negative	8 (22%)
Conventional testing, not available	2 (5%)
Clinical impact	
Positive	6 (16%)
Negative	0 (0%)
None	30 (81%)
Indeterminant	1 (3%)

mNGS, metagenomic next-generation sequencing.

**Table 2.** Cases with positive clinical impact of metagenomic next-generation sequencing

Age sex	Comorbidities	Clinical diagnosis before mNGS	Results of mNGS	Did mNGS change management?	Details	Impact
43 M	None	Pulmonary TB was clinically suspected, although AFB smear and PCR on sputum were negative multiple times	*Mycobacterium tuberculosis*	Y	The first ID attending started empiric RIPE therapy for clinically suspected TB. However, patient developed transaminase elevation. The subsequent ID attending stopped RIPE because of no confirmation of TB. mNGS came back positive and RIPE were re-started. One month later, sputum culture grew TB.	Positive
86 M	Myelodysplastic syndrome, lung cancer	Respiratory failure with lung nodule concerning for fungal infection. *E. coli* bacteremia, HSV stomatitis	HSV-1, *Aspergillus flavus*, *E. coli*, *Aspergillus fumigatus*, *Staphylococcus haemolyticus*, *Staphylococcus epidermidis*, *Bacteroides vulgatus*	Y	Deescalated from amphotericin to isavuconazole given no mucormycosis or other non-aspergillus mold. However, most organisms detected in mNGS were considered as colonization or contamination.	Positive
43 M	End stage pulmonary fibrosis requiring lung transplant	Valvular echodensity concerning for infectious endocarditis	Negative	Y	Low suspicion for endocarditis given absence of signs and symptoms of infective endocarditis. Negative Karius test result was used as supportive data. Patient was cleared for lung transplantation	Positive
17 F	Myelodysplastic syndrome status post stem cell transplant	*Granulicatella* and *Rothia* bacteremia, respiratory failure without clear etiology	*Aspergillus flavus*, *Rothia mucilaginosa*, HHV7	Y	Liposomal amphotericin-B was started for probable invasive pulmonary aspergillosis	Positive
59 M	IgA nephropathy status post renal transplant	Diffuse rash with lung infiltrates of unclear etiology	JC Virus, EBV, HPV	Y	Supported reduction in immune suppression for management of viral infection. All other infectious disease-related investigations, including skin biopsy culture, blood culture, fungal serology, TB, syphilis, and HIV workup, yielded negative results. Histopathology indicated nonspecific panniculitis. Symptoms improved following the reduction in immunosuppression.	Positive
65 M	Myelofibrosis	Lymph node biopsy conducted for cancer work up incidentally showed necrosis with AFB-positive organism. No culture was obtained.	*Mycobacterium avium*	Y	Started empiric treatment once Karius result came back positive for *mycobaceirum avium*. Blood culture was done after Karius came back positive, which confirmed disseminated *Mycobacterium avium* infection.	Positive

AFB, acid-fast bacilli, mNGS: metagenomic next-generation sequencing, TB, tuberculosis, HSV, herpes simplex virus, HHV, human herpesvirus, EBV, Epstein-Barr virus, LFT, liver function test, ID, infectious diseases, JC virus, JC polyomavirus virus, HIV, human immunodeficiency virus, HPV, human papillomavirus, CMV: cytomegalovirus.

In two of the six patients for whom the Karius test had a positive clinical impact, mycobacterial pathogens were identified, which led to the initiation of empiric therapy faster than would have been possible with AFB cultures (Table 2). In one case, the test result prompted the initiation of antifungal therapy for probable pulmonary aspergillosis. In another patient with a diffuse rash and pulmonary infiltrates, the mNGS results were positive for JC virus, Epstein-Barr virus, and human papillomavirus, supporting the reduction of immunosuppression. Antifungal therapy was de-escalated based on a negative result in another patient. In one case where a valvular echodensity was observed on echocardiography, with no risk factors or clinical stigmata of infective endocarditis, a negative result provided reassurance to move forward with lung transplantation. It is noteworthy that one of the 30 patients categorized as having ‘no impact’ based on the criteria used was clinically considered inaccurate. This patient had been experiencing renal and pulmonary lesions for months, along with a cardiac mass and a positive beta-D-glucan test result. This patient had been on antifungal therapy with mold-active agents for an extended period. Despite the Karius test returning a negative result, tissue cultures and blood cultures eventually grew *Fusarium* sp. These results, taken together with the identification of fungal hyphae in a biopsy of the cardiac mass, prove the Karius result was a false negative.

## Discussion

We conducted a manual chart review of 37 patients with Karius testing to assess the impact of metagenomic cell-free DNA testing on patient care using previously established criteria from the literature. We considered 16% to have a positive clinical impact, while 81% had no impact, and 3% had an indeterminate impact.

The positive impact rate of 16% in our patients is similar to previous reviews which demonstrated an impact of 7.3%,^4^ however is lower than others which demonstrated a positive impact of >40%.^5,6^ The variability in reported clinical impact across the literature may be attributed to the use of nonstandard criteria for classifying clinical impact. Notably, some studies classify a test that confirms a previously known diagnosis as having a positive clinical impact while the criteria we used did not. In our review, most cases in which the diagnosis was confirmed were determined to have no impact on patient care. Furthermore, variations in practices among different institutions, including differences in the timing of testing (early vs. late) and selection criteria for employing mNGS, could also contribute to differences in clinical impact.

Interestingly, in two out of the six patients with a positive clinical impact, mNGS identified a mycobacterial pathogen more rapidly than conventional testing. The test not only provided faster results than conventional cultures but also yielded a positive result for disseminated *Mycobacterium avium* complex in myelofibrosis patient and *M. tuberculosis* in one other case, despite negative sputum smears and GeneXpert MTB/RIF PCR results. This suggests that patients suspected of having slow-growing pathogens, such as *Mycobacteria* species, may be a subgroup in which mNGS is more likely to have a positive impact. Further studies with a larger sample size are necessary to confirm this observation.

Additionally, in another two of the six patients with a positive clinical impact, a clinical decision was made based on the negative results of mNGS. However, extreme caution should be exercised when utilizing a negative test result, especially in patients with a high pretest probability of infection. There is a wide range of reported sensitivity for mNGS testing in the literature (51–90%).^1,2^ We also identified one case in the ‘no impact’ group where a patient with a high burden of disease, specifically *Fusarium* sp. growing directly within the bloodstream, returned a negative mNGS test result.

The outcomes of mNGS require meticulous interpretation, irrespective of a positive or negative outcome. A positive outcome may indicate colonization or contamination, while a negative test cannot guarantee the absence of infection in a patient. It might be prudent for hospitals to limit the authorization of test orders to ID physicians or microbiologists exclusively. In this present study, the majority of patients underwent ID consultations prior to mNGS submission. This underscores the crucial contribution of infectious disease expertise in ensuring responsible test ordering, nuanced interpretation of mNGS results, and providing direction for subsequent management decisions.

In conclusion, our retrospective study found that mNGS had a positive impact on 16% of the patients for whom it was performed. While mNGS results may provide valuable supplementary information, it is essential to interpret them within the broader clinical context and evaluation. mNGS results could be beneficial in selected cases, particularly when conventional testing, such as for *Mycobacterium* infection, has a prolonged turnaround time. Prospective studies are needed to better determine the clinical scenarios in which mNGS has the greatest potential to improve patient outcomes.

## Supporting information

Olthoff et al. supplementary material Olthoff et al. supplementary material

## Supplementary material

For supplementary material accompanying this paper visit http://doi.org/10.1017/ash.2024.31. click here to view supplementary material
