# Supplementary material for: Impact of metagenomic next-generation sequencing on clinical decision-making at an academic medical center, a retrospective study, Iowa, 2020–2022
Source: Antimicrob Steward Healthc Epidemiol. 2024 Mar 27;4(1):e39. doi: 10.1017/ash.2024.31 (PMC10983051; doi:10.1017/ash.2024.31)
Supplement: Olthoff et al. supplementary material [file S2732494X24000317sup001.docx]

**Supplementary Figure 1: Criteria for determining clinical impact by Hogan et al.** ^4^

| Category of clinical impact | Change in management | Clinical impact type | Our patients, n=37 |
| --- | --- | --- | --- |
| Positive | Yes | New diagnosis based on Karius result and not confirmed by conventional microbiological methods | 1 (3%) |
|  |  | Earlier diagnosis based on Karius result and later confirmed by conventional microbiological methods | 2 (5%) |
|  |  | Karius result enabled avoidance of invasive surgical biopsy | 0 |
|  |  | Karius result enabled initiation of appropriate therapy | 0 |
|  |  | Karius result enabled de-escalation of therapy | 0 |
|  |  | Karius result enabled escalation of therapy | 3 (8%) |
|  | No | Karius result confirmed clinical diagnosis | 0 |
| Negative | Yes | Karius result led to unnecessary treatment | 0 |
|  |  | Karius result led to additional unnecessary diagnostic investigations | 0 |
|  |  | Karius result led to longer length of stay | 0 |
| None | No | Karius result showed new organism but result not acted upon | 14 (38%) |
|  |  | Karius result confirmed conventional microbiological diagnosis and not acted upon | 5 (14%) |
|  |  | Karius test result was negative and not acted upon | 10 (27%) |
|  |  | Patient died before Karius result available | 1 (3%) |
| Indeterminate | Yes | Could not determine clinical impact from chart review | 1 (3%) |
|  | No |  |  |
|  | Indeterminate |  |  |
